# Magnetic Fe_3_O_4_@SiO_2_ study on adsorption of methyl orange on nanoparticles

**DOI:** 10.1038/s41598-023-50368-x

**Published:** 2024-01-12

**Authors:** Heng Li, Huanhuan Jin, Ranran Li, Junpeng Hua, Zhen Zhang, Ruixin Li

**Affiliations:** School of Water Conservancy and Civil Engineering, Northeast Agricultural University, Harbin, 150030 China

**Keywords:** Environmental sciences, Hydrology, Nanoscience and technology

## Abstract

Magnetic core–shell Fe_3_O_4_@SiO_2_ nanoparticles were synthesized by sol–gel method. Based on the characterization and experimental results, the adsorbent was found to have an average particle size of approximately 120 nm, a pore size range of 2–5 nm and superparamagnetic properties. It exhibited electrostatic and hydrogen bonding interactions during adsorption of methyl orange (MO). The adsorption of MO on the magnetic Fe_3_O_4_@SiO_2_ nanoparticles exhibited pseudo-second-order kinetics, the adsorption process is a spontaneous endothermic adsorption process, which conforms to the Langmuir adsorption isotherm model. he maximum amount of MO was adsorbed at pH = 2, T = 45 °C and t = 30 min, and the highest adsorption capacity was 182.503 mg/g; The unit adsorption capacity of the Fe_3_O_4_@SiO_2_ nanoparticles still reached 83% of the original capacity after 5 cycles, so the material was reusable and met the requirements of environmental protection. This study reveals the great potential of magnetic mesoporous nanoparticles for removal of dyes from wastewater.

## Introduction

Organizational dyes are widely used in the textile, leather, printing, and cosmetic industries, and these industries produce large amounts of dye wastewater every year, which enters the surrounding ecosystem and causes various adverse consequences while posing serious threats to both human health and the aquatic environment^1–3^. Methyl orange (MO) is a typical anionic dye^4,5^, and the need for reasonable wastewater treatment processes is particularly important because of the complex structure of methyl orange (MO), which is not easily biodegradable or photodegradable. Various wastewater treatment technologies have received increasing attention, and the current methods used in treating wastewater include photodegradation^6^, electrochemical methods^7^, biological methods^8^, precipitation, filtration and oxidation^9^; these all have high operating costs and poor adsorption capacities, and they generate waste causing secondary pollution. Adsorption^10^ is one of the most effective dye removal methods, and adsorption is a simple, efficient, green and recyclable treatment technology that is widely used in wastewater treatment. Among the adsorbents used are chitosan^11^, zeolite^12^, activated carbon^13^, cellulose aerogel^14^ etc., but they have poor selectivities, high costs, low reuse rates and poor adsorption capacities. Nanosorbents^15^ have received much attention due to their large specific surface areas, abundant active groups or active atoms on their surfaces, high mechanical and thermal stabilities, abundant adsorption sites and high adsorption capacities, but they are also difficult to regenerate and separate.

Magnetic nanoparticles with their small particle sizes, large surface areas and unique magnetic separation properties have solved the difficult separation problems of nanosorbents and are increasingly widely used. However, bare magnetic nanoparticles exhibit poor particle dispersions and stabilities, and they are prone to agglomerate. In addition, they are easily oxidized in air or acidic solutions, which reduces the adsorption performance^16^. To solve these problems, modifications have applied to the magnetic nanomaterials. These modifications involve two process, chemical modification and surface covering, and surface covering is most commonly used. Using Fe_3_O_4_ as the magnetic core and covering the surface to prepare nanomaterials with core–shell structures improves the stability and dispersion of magnetic Fe_3_O_4_ nanoparticles, and modifications of the protective shell can also extend the range of applications for the adsorbent^17^. Mesoporous silica is often used to encapsulate the magnetic nanomaterials due to the many silica hydroxyl groups present on the surface and the high specific surface areas, large pore sizes, and biocompatibilities^18^, and the absence of redox reactions on the nanoparticle surfaces make it the preferred stabilizer^19^. Lee et al^20^.modified Fe_3_O_4_ nanoparticles with TEOs for adsorption of organic dyes from water. There are many synthetic routes to mesoporous silica, of which sol–gel chemistry^21^ is most commonly used due to the mild reaction conditions, low cost and homogeneity of the resulting product^22^.

Therefore, in this study, the SiO_2_-coated core–shell structured magnetic nanocomposite material Fe_3_O_4_@SiO_2_ was synthesized via the sol–gel method and used to adsorb methyl orange (MO) from solution. The constructed magnetic nanocomposites have magnetic dipole interactions that inhibit agglomeration, facilitate dispersion of the nanoparticles in liquids and preclude corrosion in acidic environments, so the new composites have the advantages of both magnetic nanoparticles and mesoporous silica^23^. The adsorption properties of the synthesized Fe_3_O_4_@SiO_2_ nanoparticles were investigated by XPS, XRD, TEM, SEM, VSM, Zeta and BET characterizations to determine the factors that affect the adsorption properties, such as the temperature and reaction time, and then the kinetic model, isothermal adsorption model, internal diffusion model, and adsorption thermodynamics were used to analyze the mechanism and performance of the reaction. Finally, we found that Fe_3_O_4_@SiO_2_ nanoparticles exhibited excellent adsorption of MO, which lays a foundation for preparation of magnetic nanomaterials designed to adsorb MO from wastewater.

## Materials and methods

### Materials

Methyl orange (C_14_H_14_N_3_SO_3_Na), ferric chloride hexahydrate (FeCl_3_·6H_2_O), concentrated ammonia (NH_3_·H_2_O), methanol (CH_3_OH), ethyl orthosilicate (TEOS), polyethylene glycol 4000 (PEG4000), powdered ferric tetroxide (Fe_3_O_4_), hydrochloric acid (HCl), and sodium hydroxide (NaOH) were purchased from Sinopharm Chemical Reagent Co, Ltd. and were analytically pure. The water used in the experiments was deionized water.

### Magnetic mesoporous silica nanoparticles

#### Synthesis of Fe_3_O_4_ nanoparticles by chemical precipitation

FeCl_3_·6H_2_O (16 g) was added to 100 mL of water and poured into a conical flask, and then 12.8 g of Fe_3_O_4_·7H_2_O was added, shaken and split into two iron solutions. Then, 0.1 mol/L PEG-4000 and 6 mol/L NH_3_·H_2_O solutions were prepared. Twenty-five milliliters of PEG4000 solution and 25 mL of 6 mol/L NH_3_·H_2_O were added to the four flasks under a N_2_ atmosphere with stirring, and after 10 min, the two iron solutions were added to the four flasks with a peristaltic pump at a flow rate of 0.1 L/h. The solutions were allowed to react for 5 min, and the magnetic nanoparticles were obtained by warming at 60 °C for 1 h in a constant temperature water bath and adding deionized water after maturation at 80°C for 0.5 h^24^. The reaction is shown in Eq. 1:

1 $$ {\text{Fe}}^{2 + } + 2{\text{Fe}}^{3 + } + 8o{\text{H}}^{ - } = {\text{Fe}}_{3} {\text{O}}_{4} \downarrow + 4{\text{H}}_{2} {\text{O}}^{{}} $$

#### Preparation of Fe_3_O_4_@SiO_2_

**Figure 1:**
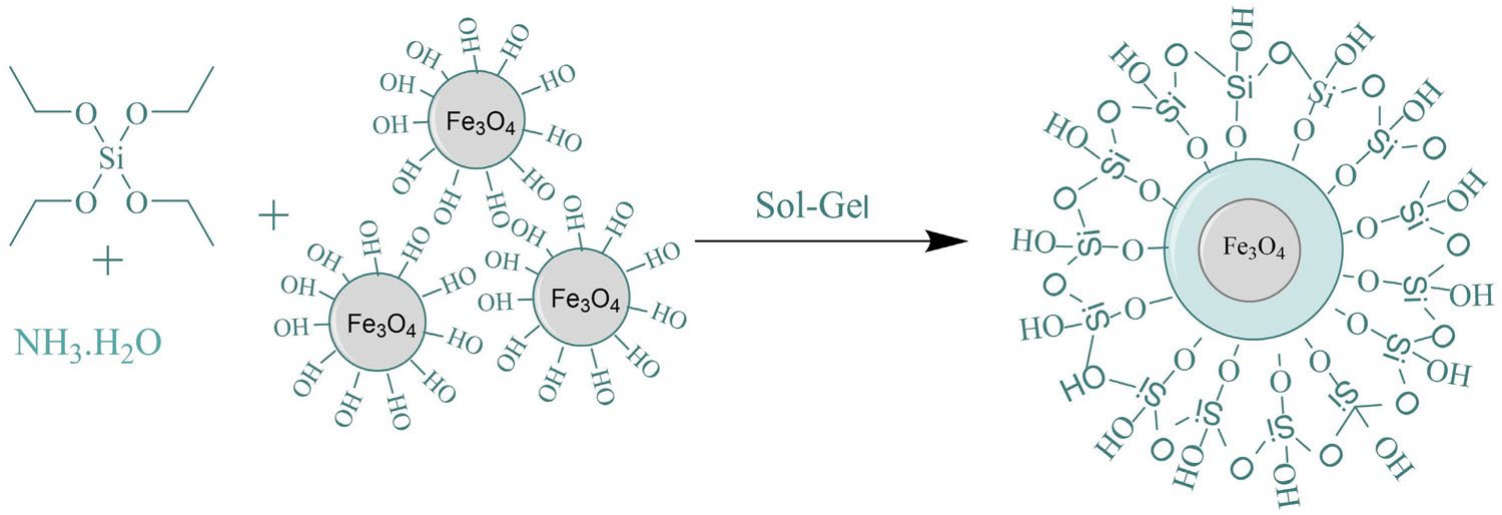
Synthetic process for Fe_3_O_4_@SiO_2_.

In this experiment, Fe_3_O_4_@SiO_2_ was prepared via the sol–gel method, and 2 g of the wet nanoparticles were sonicated in 40 mL of water. Since ethyl orthosilicate (TEOS) produces SiO_2_ under base catalysis, TEOS was used to introduce the silica groups. TEOS (1.76 mL) was sonicated in 80 mL of methanol, and the two previous solutions were added to a four-necked flask and sonicated for 15 min. Then, 4 mL of NH_3_·H_2_O was added, sonicated for 15 min and stirred for 4 h. After stirring, the magnetic material was separated, washed with methanol and deionized water until neutral, and soaked in HCl at pH = 1 for 24 h to remove the excess silicon coating on the Fe_3_O_4_@SiO_2_ surface. Finally, Fe_3_O_4_@SiO_2_ nanoparticles were obtained after washing with methanol and water to neutral and drying in a freeze-dryer for 24 h. The reaction is shown schematically in Fig. 1.

### Characterization

The crystal structures of the prepared materials were analyzed by X-ray diffraction (XRD, Panalytical X'Pert Pro, NL). Transmission electron microscopy (TEM, FEI Tecnai F20, US) and scanning electron microscopy (SEM, JEOL JSM-7401F, US) were used to analyze the surface morphologies and microscopic features of the prepared materials. Fourier infrared spectroscopy (FTIR, Thermo Scientific Nicolet iS20, US) was used to determine the main chemical components of the materials. The magnetic properties were determined with the VSM hysteresis line test (VSM, LakeShore7404, US). The specific surface areas, pore sizes and pore volumes of the material were determined with a fully automated specific surface and porosity analyzer (BET, Micromeritics ASAP 2460, US). X-ray electron spectroscopy (XPS, Thermo Scientific K-Alpha, US) was used to detect the chemical elements and electronic states of the prepared materials. The zeta potential analysis and particle size analysis of adsorbents were carried out by Malvern Zetasizer Nano ZS90 (Malvern Instruments Ltd.). Before testing, it is necessary to adjust the pH of MO (50ml) solution to 2, 4, 6, 8, and 10 by using 0.1M HCl and 0.1M NaOH, and add them to each solution 20mg Fe_3_O_4_@SiO_2_ Ultrasound for 30 min.

### Adsorption experiments

In this paper, MO adsorption with different influencing factors was investigated by UV‒visible spectrophotometry. Methyl orange solutions are commonly used as an acid‒base indicator, and the absorbance changes at different pHs. In this study, the absorbance of methyl orange was measured at different pHs, and it was found that the absorbance changes were very small and could therefore be neglected. A spectral scan of MO revealed that the maximum adsorbance occurred 464 nm, so 464 nm was used as the wavelength of MO in these experiments. Under the condition of room temperature 20 ℃, 1mg/L, 5mg/L, 10mg/L, 15mg/L, 25mg/L, 50mg/L concentration of MO solution was selected to measure the absorbance, and the MO concentration was taken as the horizontal coordinate, absorbance as the vertical coordinate to make the standard curve, and the curve was made a univariate linear regression curve to get the standard curve: y = 0.0772x + 0.0007, in which R^2^ was 0.9997, after comprehensive consideration, the concentration of the absorbance measurement was uniformly diluted to 2–5 mg/L, to reduce the influence of the reduction of solution volume on the experiment. 0.9997, after comprehensive consideration, the concentration of absorbance measurement in the experiment was uniformly diluted to 2–5 mg/L, to reduce the impact of the reduction of the amount of solution on the experiment.

The effects of the initial concentration, pH, reaction time and temperature on MO adsorption were investigated. For the pH adsorption study, the pH ranged from 2 to 10, the MO concentration was 100 mg/L, different pH were established with 0.1 mol/L HCl and NaOH solutions, the temperature was 298 K, and samples were taken at different times within 150 min. For the reaction time adsorption study, the initial concentration of MO was 100 mg/L, and the reaction time was 150 min with samples taken at different times. For studies of the initial MO concentration and temperature, 100 mL solutions with different concentrations ranging from 10 to 120 mg/L were kept at 298 K, 308 K and 318 K, and the reaction times were 30 min. The national standards (GB 8978-8, GB 18,918–202) indicate that the allowed pH range for discharged waste solutions is 6–9. In this study, the pH of the 100 mg/L MO solution was approximately 6.43 at room temperature, which was consistent with the emission standard, so there was no need to adjust the pH when investigating influencing factors other than the pH. Different amounts of the simulated MO wastewater were added to 150 mL conical flasks with 25 mg of adsorbent and then shaken in a constant temperature water bath shaker, and the supernatant was taken after shaking for an appropriate period to determine the absorbance A at 464 nm. The MO concentrations were measured photometrically with an ultraviolet spectrophotometer(UV, AIpha1506,CHN).

The absorbance A and a standard curve were used to calculate the concentration of methyl orange, and then the amount adsorbed, η_e_ (Eq. 2), and removal rate, Q_e_ (Eq. 3), were calculated with the following equations based on the change in the MO concentration after adsorption:

2 $$ \eta_{{\text{e}}} = \frac{{{\text{C}}_{0} - {\text{C}}_{e} }}{{{\text{C}}_{{0}} }} \times 100\% $$

3 $$ {\text{Q}}_{{\text{e}}} = \frac{{{\text{C}}_{0} - {\text{C}}_{e} {\text{V}}}}{{\text{m}}} $$

where Q_e_ (mg/g) is the unit amount of MO solution adsorbed, C_0_ (mg/L) is the initial concentration of the MO solution, C_e_ (mg/L) is the final concentration of the MO solution, V (L) and m (g) are the volume and mass of the MO solution, and η_e_ is the removal rate of the MO in solution.

### Regeneration study

To investigate the reusability of the Fe_3_O_4_@SiO_2_ nanomaterial, a 0.5 mol/L NaOH solution was used as the desorption solution because the adsorbent surface was negatively charged under strongly alkaline conditions, and the amount of MO adsorbed decreased sharply, which facilitated desorption. The Fe_3_O_4_@SiO_2_-MO material was placed in a 0.5 mol/L NaOH solution, oscillated in a constant temperature water bath for 1 h, freeze-dried after magnetic separation, and cross-washed with methanol and deionized water 3 times. The adsorption process was performed again with T = 25 °C, t = 30 min, and C_0_ = 100 mg/L, and the adsorption–desorption cycle was repeated 5 times.

### Real sample preparation

In order to verify the adsorption capacity of Fe_3_O_4_@SiO_2_ in real water samples, precipitation samples were collected from Fujin City (FJ) and Kedong (KD) County, Heilongjiang Province, respectively. Before use, all water samples were filtered through a 0.45 μm membrane filter (Tianjin Jinteng Instrument Factory, Tianjin, China). Experimentally, 100 mL of water sample was selected for each point sample, and 0.1 g of MO was added to formulate a 100 mg/L MO solution, and 20 mg of Fe_3_O_4_@SiO_2_ was added, respectively, and the unit adsorption and desorption rates were measured after 30 min.

### Ethics approval 

This work does not contain any research involving humans or animals. 

### Consent to participate 

The work described has not been published before; that it is not under consideration for publication anywhere else; that written informed consent was obtained from individual or guardian participants. 

### Consent to publish 

The publishment consent was obtained from all co-authors.

## Results and discussion

### Characterization

**Figure 2:**
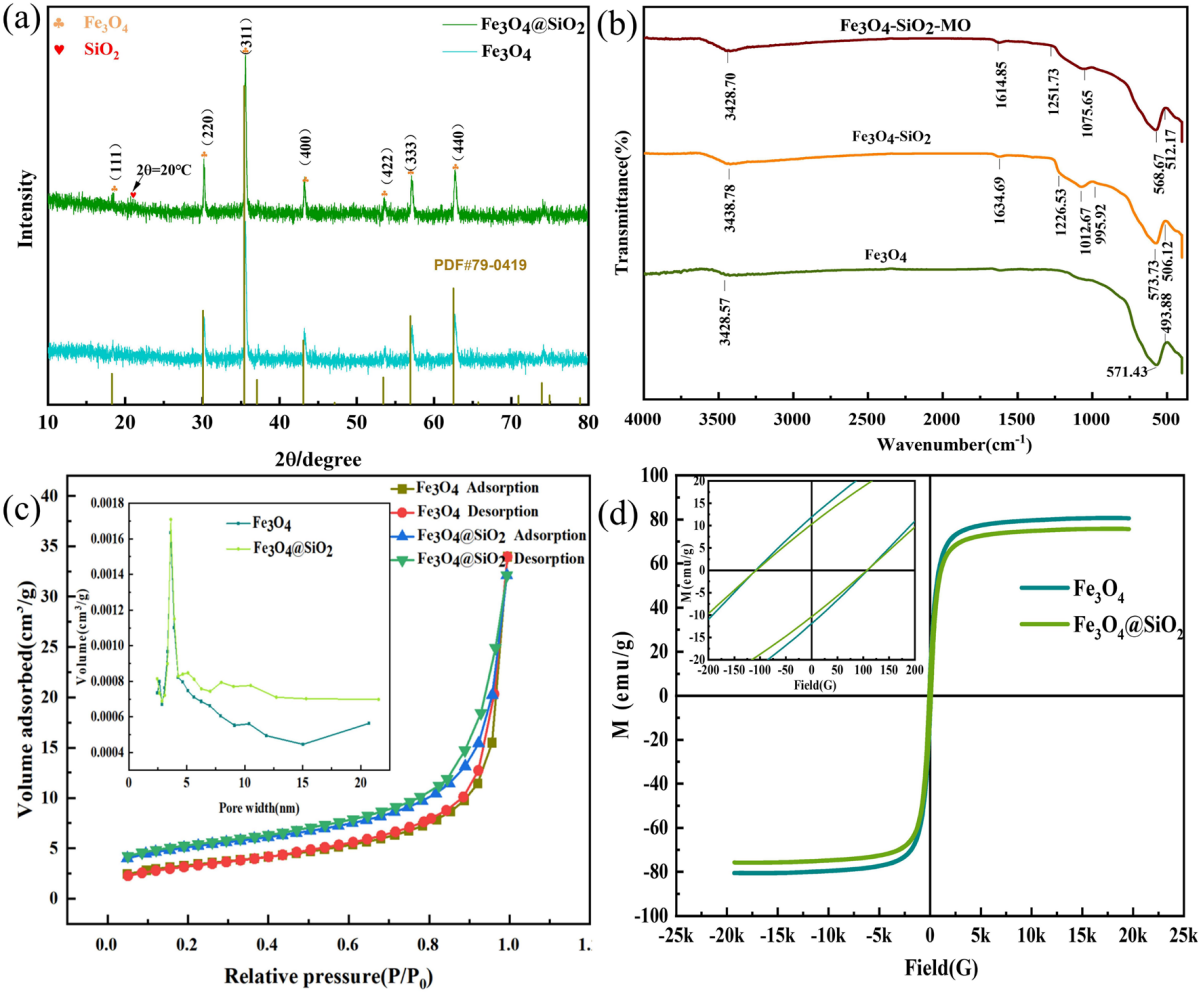
(**a**) XRD patterns for Fe_3_O_4_ and Fe_3_O_4_@SiO_2_; (**b**) IR spectra of Fe_3_O_4_, Fe_3_O_4_@SiO_2_, and Fe_3_O_4_@SiO_2_-MO; (**c**) adsorption–desorption isotherms of Fe_3_O_4_ and Fe_3_O_4_@SiO_2_ and the pore size distributions; (**d**) VSM diagrams for Fe_3_O_4_ and Fe_3_O_4_@SiO_2_.

Figure 2a shows the XRD patterns for the adsorbents Fe_3_O_4_ and Fe_3_O_4_@SiO_2_. As seen from the figure, the diffraction peaks at 30.1°, 35.4°, 43.1°, 53.4°, 56.9°, and 62.5° corresponded to the standard pattern (PDF#79-0419) and the (220), (311), (400), (422), (511), and (440) crystalline planes of the cubic Fe_3_O_4_ nanoparticles; there were no impurity peaks, such as those for FeO and Fe_2_O_3_, proving that the resulting product was single-phase cubic crystalline Fe_3_O_4_. Additionally, the XRD pattern for Fe_3_O_4_@SiO_2_ showed the same crystalline planes as Fe_3_O_4_, but the peak intensities were lower, indicating that the crystalline structure of Fe_3_O_4_ was not changed and was successfully encapsulated. A broad absorption peak appeared at 2θ = 20° for amorphous SiO_2_, proving that SiO_2_ was successfully coated on Fe_3_O_4_ and that the composite material Fe_3_O_4_@SiO_2_ was prepared successfully.

To determine the material structure and the functional groups of Fe_3_O_4_, Fe_3_O_4_@SiO_2_ and Fe_3_O_4_@SiO_2_-MO, they were analyzed by FT-IR, and the results are shown in Fig. 2b. The figure shows that the three adsorbents exhibited absorption bands near 570 cm^−1^ for the Fe–O stretching vibration^25^, and the stretching vibration band for –OH appeared at 3428.57 cm^−1^. The Fe_3_O_4_@SiO_2_ absorption band at 995.92 cm^−1^ was a bending vibrational absorption band for Si–O–H. The bending vibrational absorption band of Si–OH appears near 1634.69 cm^−1^ .The strong Si–O–Si antisymmetric stretching vibrational band at 1072.67 cm^−1^ indicated that silica had successfully encapsulated the Fe_3_O_4_ particles^26^. The characteristic peaks for the surfactant PEG-4000 did not appear because the surfactant was removed during several washes with methanol and deionized water before freeze-drying. Therefore, the characteristic peaks for Fe_3_O_4_ were still present after introduction of the SiO_2_ shell as well as adsorption of MO from solution, and the main difference was the introduction of SiO_2_ characteristic peaks.

Additionally, the Fe–O stretching vibration band of Fe_3_O_4_ shifted from 571.43 to 573.43 cm^−1^, probably due to the formation of new Si–O–Fe bonds during the reaction in which Si combined with Fe through the O atom to form a new complex. In contrast to Fe_3_O_4_@SiO_2_, the Fe_3_O_4_@SiO_2_-MO spectrum showed a shift of the OH stretching band from 3438.38 to 3448.98 cm^−1^, indicating that MO underwent hydrogen bonding and formed electrostatic interactions with Fe_3_O_4_@SiO_2_^27^.

N_2_ adsorption–desorption is often used with mesoporous substances to determine the nature and surface areas of the pores. BET measurements were performed with Fe_3_O_4_, Fe_3_O_4_@SiO_2_, BTT, and CAC, and the results are shown in Fig. 2c and Table 1. The figure shows that the isotherms for the Fe_3_O_4_ and Fe_3_O_4_@SiO_2_ adsorbents were type II isotherms based on the IUPAC classification, and obvious hysteresis loops appeared with increasing relative pressures, indicating that the synthesized materials were mesoporous. As seen from the figure, P/P_0_ was less than 0.6. The adsorption process was weakly affected by P/P_0_, indicating that adsorption coalescence occurred on the pore walls during this process. The adsorption capacity increased significantly when P/P_0_ was greater than 0.6, at which time the adsorption and desorption curves of Fe_3_O_4_ and Fe_3_O_4_@SiO_2_ were no longer coincident; this may have resulted from capillary coalescence, indicating that the mesopores of the two synthesized materials were small and had uniform size distributions. Based on the BJH method, most of the pore sizes of Fe_3_O_4_ and Fe_3_O_4_@SiO_2_ were concentrated^28^ in the 2–5 nm range, which indicated a mesoporous material. Table 1 shows that the pores of Fe_3_O_4_ and Fe_3_O_4_@SiO_2_ were larger than those of BTT and CAC, and the surface area, pore size and pore volume of Fe3O4@SiO2 were larger than those of Fe_3_O_4,_ implying more adsorption sites and enhanced adsorption capacity.

**Table 1:** BET parameters for Fe_3_O_4_, Fe_3_O_4_@SiO_2_, BTT and CAC.

Sample	BET area (${\text{m}}^{2} {\text{g}}^{-1}$)	Pore size (nm)	Pore volume ${\text{(cm}}^{2} \, {\text{g}}^{-1})$
Fe_3_O_4_	11.5093	7.4349	0.021998
Fe_3_O_4_@SiO_2_	17.7188	7.7382	0.029849
BTT	61.2236	6.5024	0.099525
CAC	170.0739	3.3696	0.143268

Figure 2d shows the hysteresis lines of the Fe_3_O_4_ and Fe_3_O_4_@SiO_2_ adsorbents measured at 300 K, as shown in Fig. 2a,b. The hysteresis lines were symmetric about the origin, the magnetization intensities increased rapidly with increases in the applied magnetic field, and the magnetic properties of the magnetic nanomaterials weakened rapidly until zero when the magnetic field was weakened. The hysteresis lines of the two adsorbents showed typical S-shapes, and the residual magnetization intensities tended toward zero, indicating super paramagnetism. Additionally, the saturation magnetization intensity was 75.96 emu g^−1^ for Fe_3_O_4_, and the saturation magnetization intensity was 72.08 emu g^−1^, as seen from the figure. Since the magnetic saturation intensity of the Fe_3_O_4_ nanoparticles was reduced after the outer layer was coated with SiO_2_, it still had strong magnetic separation properties and could be separated by an external magnetic field, which was used in the adsorption tests of the organic dye.

**Figure 3:**
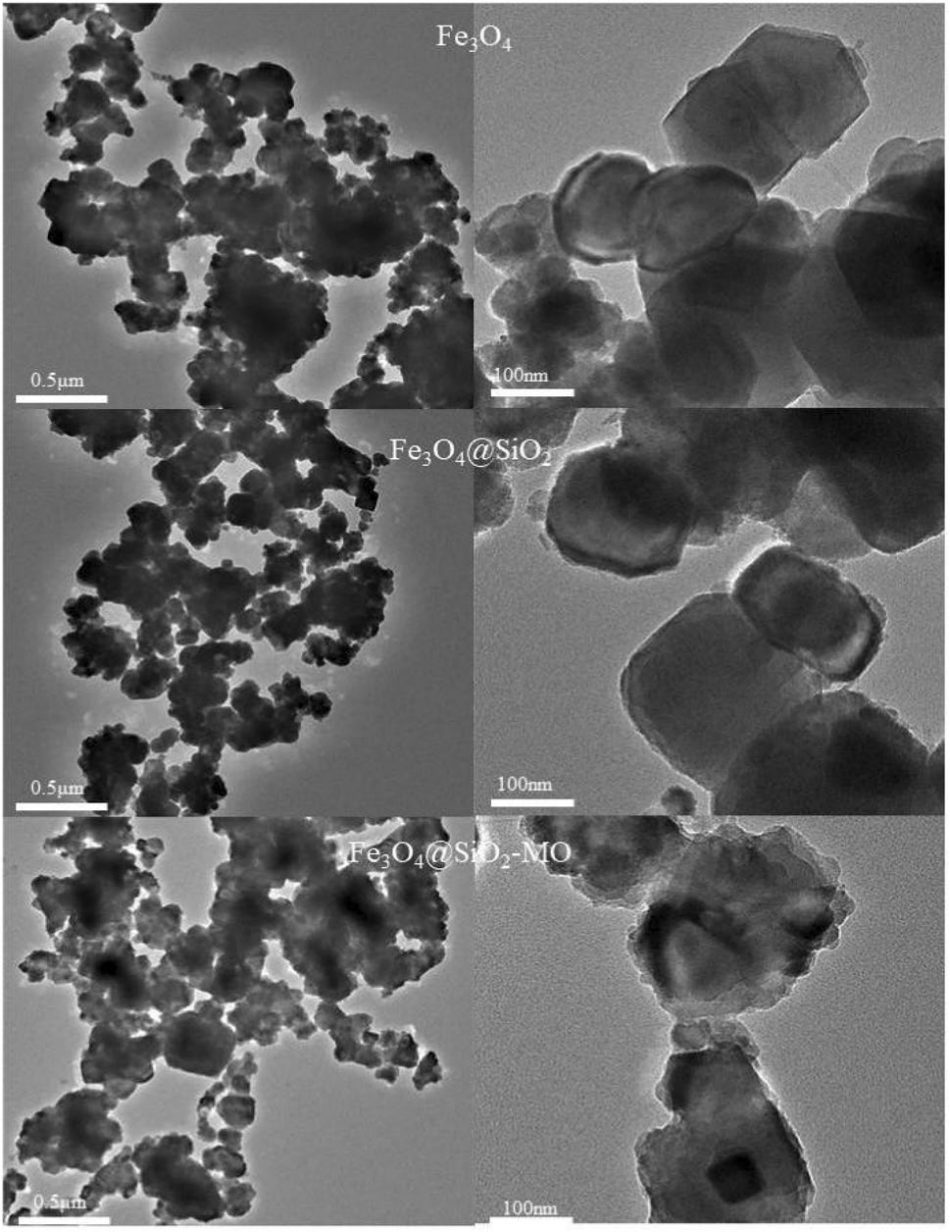
TEM images of Fe_3_O_4_, Fe_3_O_4_@SiO_2_ and Fe_3_O_4_@SiO_2_-MO.

To further confirm the XRD and FT-IR results for both adsorbents, morphological analyses of Fe_3_O_4_, Fe_3_O_4_@SiO_2_, and Fe_3_O_4_@SiO_2_-MO were performed with TEM, and the results are shown in Fig. 3 and Fig. S2. The Fe_3_O_4_@SiO_2_ nanoparticles presented spherical shapes with uniform sizes and an average particle size of approximately 100 nm, and it is clear that the Fe_3_O_4_ nanoparticles exhibited a black color encapsulated by the gray SiO_2_ shell, and the composite nanoparticles presented obvious core–shell structures, which indicated that polymer formation was successful. A comparison of the Fe_3_O_4_ and Fe_3_O_4_@SiO_2_ data showed that Fe_3_O_4_ agglomeration was significantly inhibited and the dispersion was enhanced after encapsulation by SiO_2_; the number of pores present on the surface was increased and the particle sizes were increased, so there were more adsorption sites and the adsorption performance was enhanced. A comparison of the Fe_3_O_4_@SiO_2_ images obtained before and after adsorption showed that the core–shell structure remained unchanged, but the pores were significantly reduced and showed uneven surfaces because the MO adsorbed by the Fe_3_O_4_@SiO_2_ adsorbent filled the pores but did not destroy the core–shell structure, proving that the adsorbent pore channels were highly ordered, relatively stable and not easily destroyed. In addition, both adsorbents were found to undergo agglomeration and exhibit particle size inhomogeneity.

**Figure 4:**
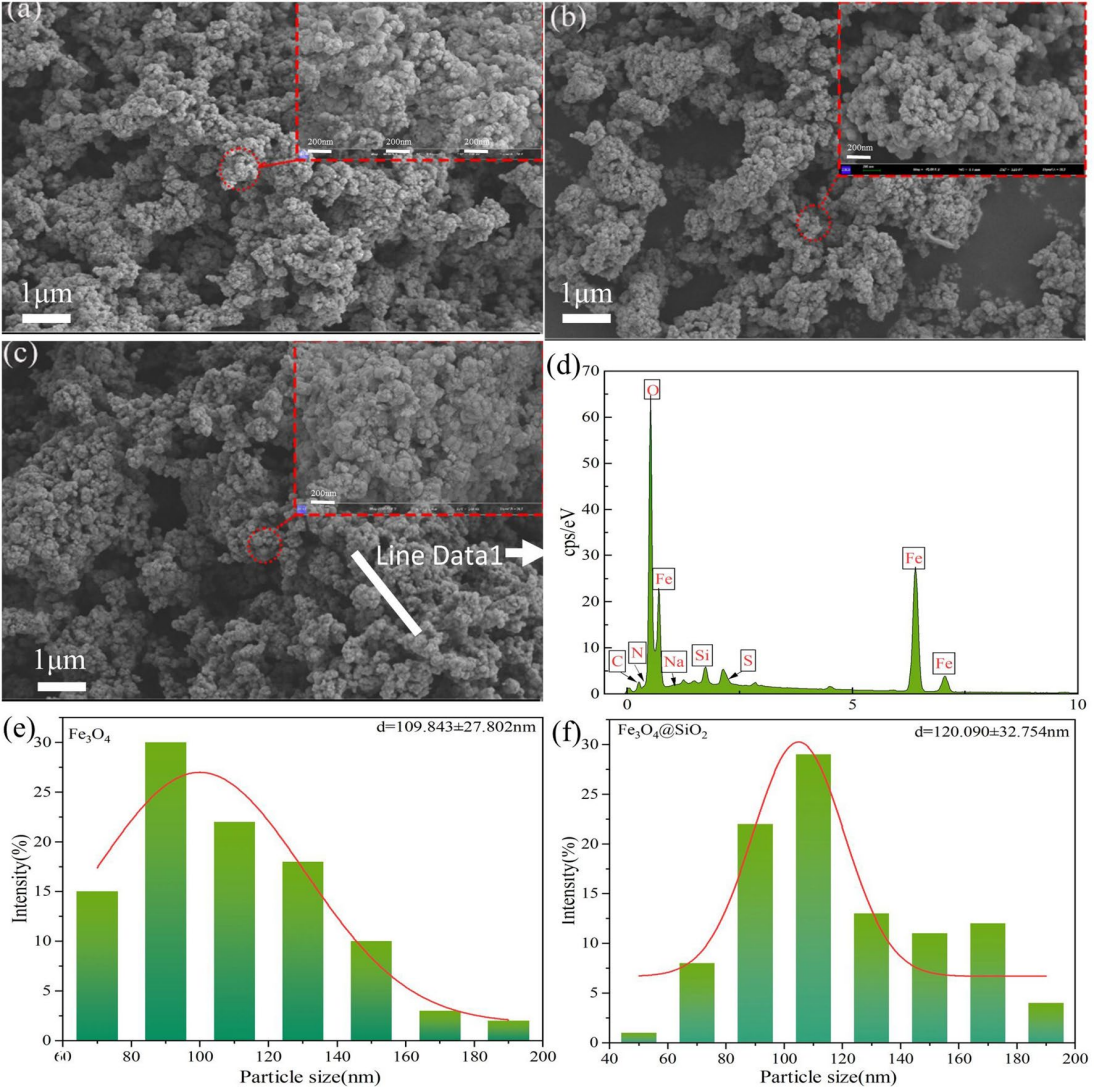
(**a**), (**b**), and (**c**) SEM images of Fe_3_O_4_, Fe_3_O_4_@SiO_2,_ and Fe_3_O_4_@SiO_2_-MO, respectively; (**d**) EDS spectra of Fe_3_O_4_@SiO_2_-MO; and (**e**) and (**f**) particle size distributions for Fe_3_O_4_ and Fe_3_O_4_@SiO_2_, respectively.

Scanning electron microscopy and energy spectrum line sweeps were performed with Fe_3_O_4_, Fe_3_O_4_@SiO_2_, and Fe_3_O_4_@SiO_2_–MO to analyze the surface morphologies and particle sizes of the synthesized products, and the results are shown in Fig. 4. In addition, the particle size statistics were generated with SEM maps and Nanomeasure particle size annotation, and the results are shown in Fig. 4e,f. The decrease in mesopores from the comparison of b and c was due to the adsorbed mass of MO in the mesopores, which was consistent with the TEM results. From Fig.S1 (a), (b) and (c), it can be seen that Fe_3_O_4_ presents a regular spherical shape, the magnetic particles have no breakage phenomenon, and the surface is clearly visible and angular, and Fe_3_O_4_@SiO_2_ still presents a spherical shape but the surface contour is more blurred. It is obvious from Fig. 4d that three peaks for S, N and Na with contents of 1.2%, 1.5% and 0.16%, respectively, appeared after the adsorption of MO by Fe_3_O_4_@SiO_2_, indicating that the MO was successfully adsorbed on the surface of Fe_3_O_4_@SiO_2_. In addition, Fig. 4e,f shows that the average particle size for Fe_3_O_4_ was 109.843 nm. The average particle size increase for Fe_3_O_4_@SiO_2_ was 120.09 nm, so the thickness of the SiO_2_ layer was approximately 10 nm, the surface area increased, the number of adsorption sites increased, and adsorption was enhanced.

XPS is commonly used to determine the elemental composition, content, chemical valence, and chemical bonding properties of materials. XPS was used to determine the surface species and adsorption mechanism for the Fe_3_O_4_@SiO_2_ mesoporous nanomaterial before and after MO adsorption.

**Figure 5:**
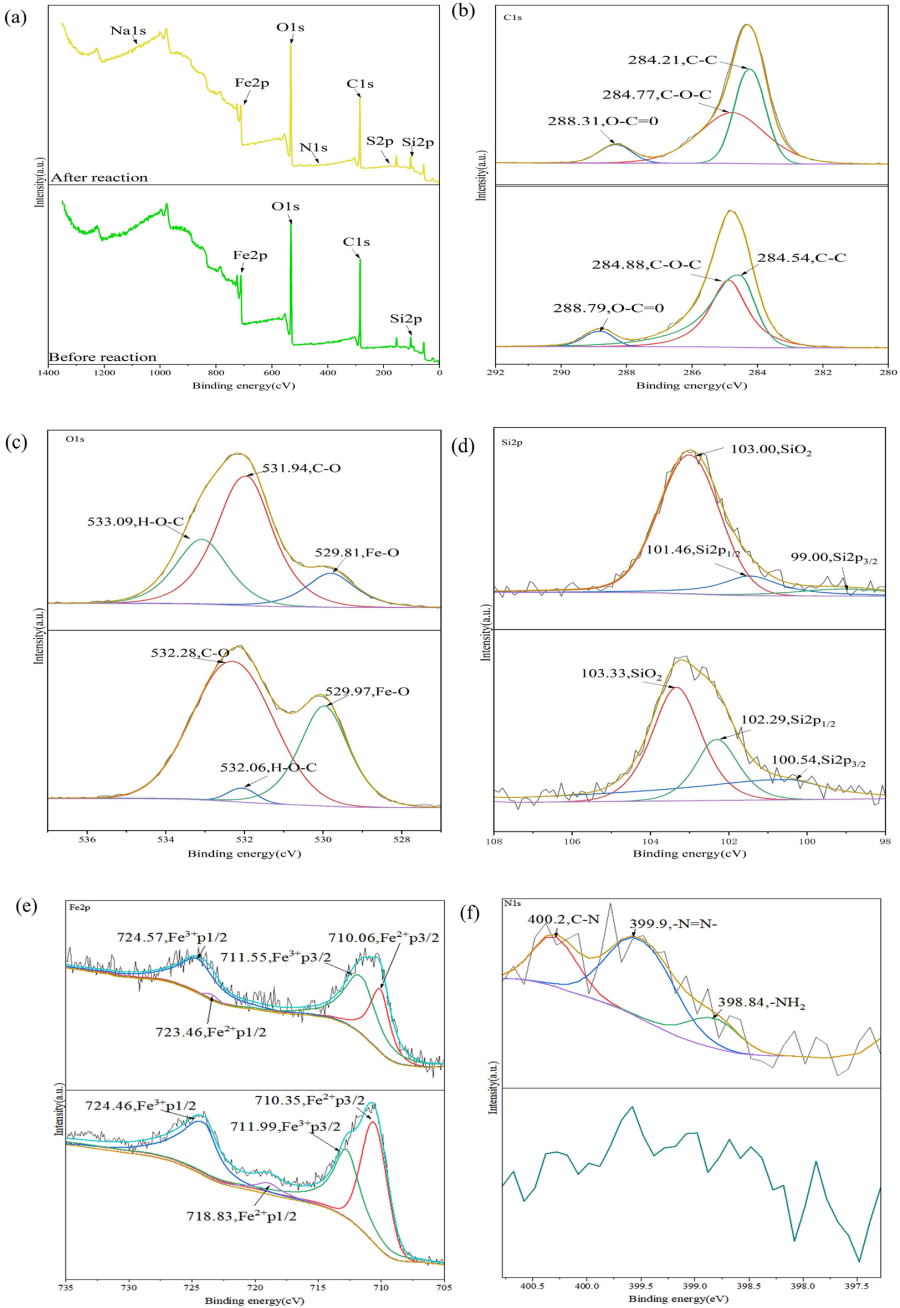
XPS spectra of Fe_3_O_4_@SiO_2_; (**a**) Total elemental spectrum; (**b**) C peaks; (**c**) Si peaks; (**d**) O peaks; (**e**) Fe peaks and (**f**) N peaks.

The XPS spectra of Fe_3_O_4_@SiO_2_ before and after adsorption are shown in Fig. 5, and the results of split peak fitting and the functional groups are shown in Table 2. Figure 5a shows that Fe_3_O_4_@SiO_2_ contained C, O, Fe, and Si elements, and after adsorption, the absorption peaks for S, Na, and N exhibited increased intensities; the atomic ratio of N 1s element was 1.32%, the atomic ratio of S2p element was 1.88%, and the atomic ratio of Na 1s element was 0.3%, and the N 1s spectrum generated the N=N absorption peak for MO at 399.90 eV. This showed that the Fe_3_O_4_@SiO_2_ nanomaterials had adsorbed the MO. The C 1s, O 1s, Fe 2p, and Si 2p binding energies were 284.80, 531.92, 710.49, and 102.73 eV, respectively, and these were obtained with a peak search of the total spectrum after correcting the binding energies with the 284.80 eV binding energy for the C 1s peak. Table 3 shows that Fe_3_O_4_@SiO_2_ had the highest C content and the lowest Fe content. The peaks at 711.99 eV and 718.83 eV corresponded to the Fe^3+^ 2p_3/2_ and Fe^2+^ 2p_1/2_ binding and moved to 711.55 eV and 723.46 eV, respectively, indicating a surface complexation reaction between Fe and O^29^. The O 1s binding energies for C–O and H–O–C changed, indicating that –OHs on the Fe_3_O_4_@SiO_2_ surface were involved in the adsorption process. Therefore, there was coordination with the adsorbent, and the adsorption mechanism included electrostatic interactions and coordination.

**Table 2:** XPS spectral parameters for Fe_3_O_4_@SiO_2_-MO.

Type	Peak position eV	Area CPS.eV	Atomic ratio %
C 1s	285.15	226,230.90	45.22
O 1s	532.09	506,692.57	43.12
Si 2p	103.19	39,928.07	8.10
Fe 2p	709.99	3176.64	0.06
Na 1s	1072.27	8906.92	0.30
S 2p	170.10	10,037.24	1.88
N 1s	400.76	11,231.03	1.32

**Table 3:** XPS spectral parameters for Fe_3_O_4_@SiO_2_.

Type	Peak position eV	Area CPS.eV	Atomic ratio %
C 1s	284.36	328,574.50	45.77
O 1s	531.92	462,224.60	43.04
Si 2p	102.73	41,336.19	9.09
Fe 2p	710.49	109,167.94	2.8

### Adsorption and desorption study

#### Effect of pH

**Figure 6:**
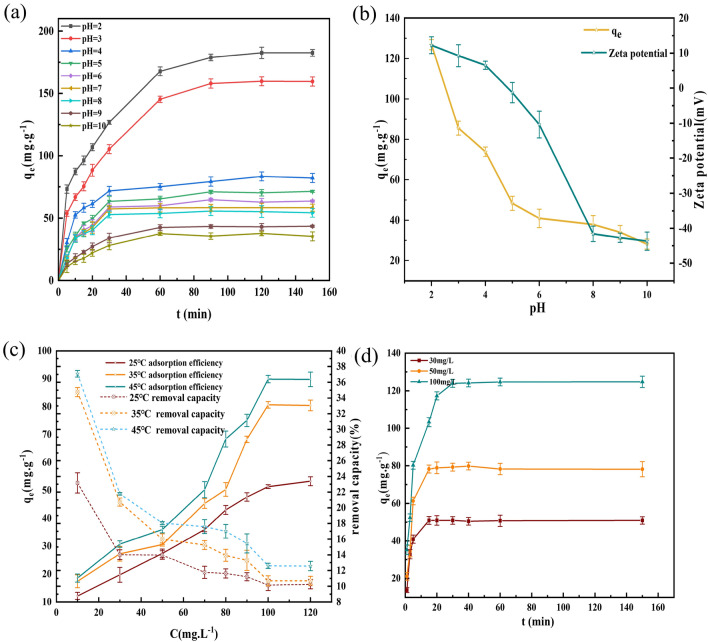
(**a**) Effect of the initial solution pH on adsorption of MO by the Fe_3_O_4_@SiO_2_ nanomaterial; (**b**) Effect of the Fe_3_O_4_@SiO_2_ zeta potential at different pH values and the effect of the pH at t = 30 min on adsorption by Fe_3_O_4_@SiO_2_; (**c**) Effects of temperature and the initial MO concentration on adsorption by the nanomaterial; (**d**) Effect of reaction time on the adsorption of MO by the Fe3O4@SiO2 nanomaterial.

As seen in Fig. 6a, the pH of the solution had a significant effect on the adsorption process. The amount of MO adsorbed by Fe_3_O_4_@SiO_2_ decreased with increasing pH, and the best adsorption effect was achieved at pH = 2, where the amount adsorbed reached 182.503 mg/g. The equilibrium amount of MO adsorbed by Fe_3_O_4_@SiO_2_ is shown in Eq. 4.

4 $$ R - OH\underset{{OH^{ - } }}{\overset{{H^{ + } }}{\rightleftharpoons}}R - OH_{2}^{ + } $$

Figure 6b Analysis of the potential maps for different pH values. The potential value of the Fe_3_O_4_@SiO_2_ nanomaterial decreased with increasing solution pH, and the equipotential point was approximately 4. When the solution pH < 4, the surface of the Fe_3_O_4_@SiO_2_ material was protonated and positively charged, which enhanced adsorption of negatively charged species, and the density of the –OH_2_^+^ ions on the adsorbent surface was gradually increased^30^. Strong electrostatic attractions occurred with the –SO_3_^−^ groups of anionic MO, which increased the adsorption efficiency. At neutral pH, the negative surface charges of the Fe_3_O_4_@SiO_2_ adsorbent were dispersed in the aqueous solution due to particle-to-particle electrostatic repulsions, and good dispersion inhibits magnetic separation and reduces the adsorption efficiency. The –OH^–^ groups present in the solution at high pH compete with the anionic MO for the positively charged adsorption sites and reduce the adsorption efficiency. In addition, the pH also affects the chemical structure of MO; when the pH < 3.4, MO exhibits a red quinone-like structure, and the sulfonic acid end of the molecule is negatively charged, which favors binding to –OH_2_^+^ on the surface, and when the pH > 3.4, MO exhibits a yellow azo structure, which is more stable and difficult to remove than the quinone-like structure^31^. The above analysis is consistent with the effect of pH on Fe_3_O_4_@SiO_2_, confirming that MO was adsorbed through electrostatic interactions.

#### Effect of adsorption time

The adsorption time is an important variable in the adsorption process, and Fig. 6d shows that the equilibrium times for adsorption of MO on the Fe_3_O_4_@SiO_2_ nanomaterial were 15 min, 20 min, and 30 min for concentrations of 30 mg/L, 50 mg/L, and 100 mg/L, respectively. At lower concentrations, the adsorption of MO on the Fe_3_O_4_@SiO_2_ nanomaterial reached equilibrium in a shorter time, and at higher concentrations, the adsorption process was more active, the adsorbent surface was blocked by dye molecules, and the required adsorption time was longer. In addition, we found that the adsorption capacity was significantly higher at the initial stage of the reaction, indicating that there were still many empty spaces on the adsorbent surface at this time, and the interaction between the MO and Fe_3_O_4_@SiO_2_ nanomaterial was not optimal at this time. As the reaction time increased, the adsorption sites on the adsorbent surface were continuously occupied by MO, the number of available adsorption sites gradually decreased, and the adsorption rate gradually decreased until the saturation point^32^.

#### Effects of temperature and initial concentration

Figure 6c shows that at the same temperature, the adsorption capacity increases continuously with increasing concentration, and the adsorption rate reaches a maximum at an MO concentration of 100 mg/L. The maximum adsorption capacity reached 89.882 mg/g at T = 45°C, while the removal rate decreased continuously. This occurred because when the MO concentration was low, there were excess adsorbent binding sites and the adsorption capacity increased significantly, but as the MO concentration increased, more adsorption sites were filled, and the adsorption capacity did not increase significantly.

Additionally, the figure shows that the adsorption and removal rates increased with increasing temperatures at the same concentration, which indicated that the adsorption of MO on the adsorbent was an endothermic process and that higher temperatures activated the adsorption sites and increased the kinetic energy for adsorption by Fe_3_O_4_@SiO_2_. Since the adsorption capacity increased with increasing temperature, it occurred via chemisorption. Conversely, if the adsorption had decreased with increasing temperature, physisorption would have been indicated ^.^ Thus, this results is consistent with the adsorption kinetics and indicated that chemisorption drove the adsorption process.

#### Adsorption kinetics

To investigate the effect of the initial concentration as well as the adsorption time, 20 mg of the Fe_3_O_4_@SiO_2_ adsorbent was added to 30, 50 and 100 mg/L MO solutions, and the absorbance was measured by sample the supernatant at after 1, 3, 5, 15, 20, 30, 40, 60 and 150 min.

**Figure 7:**
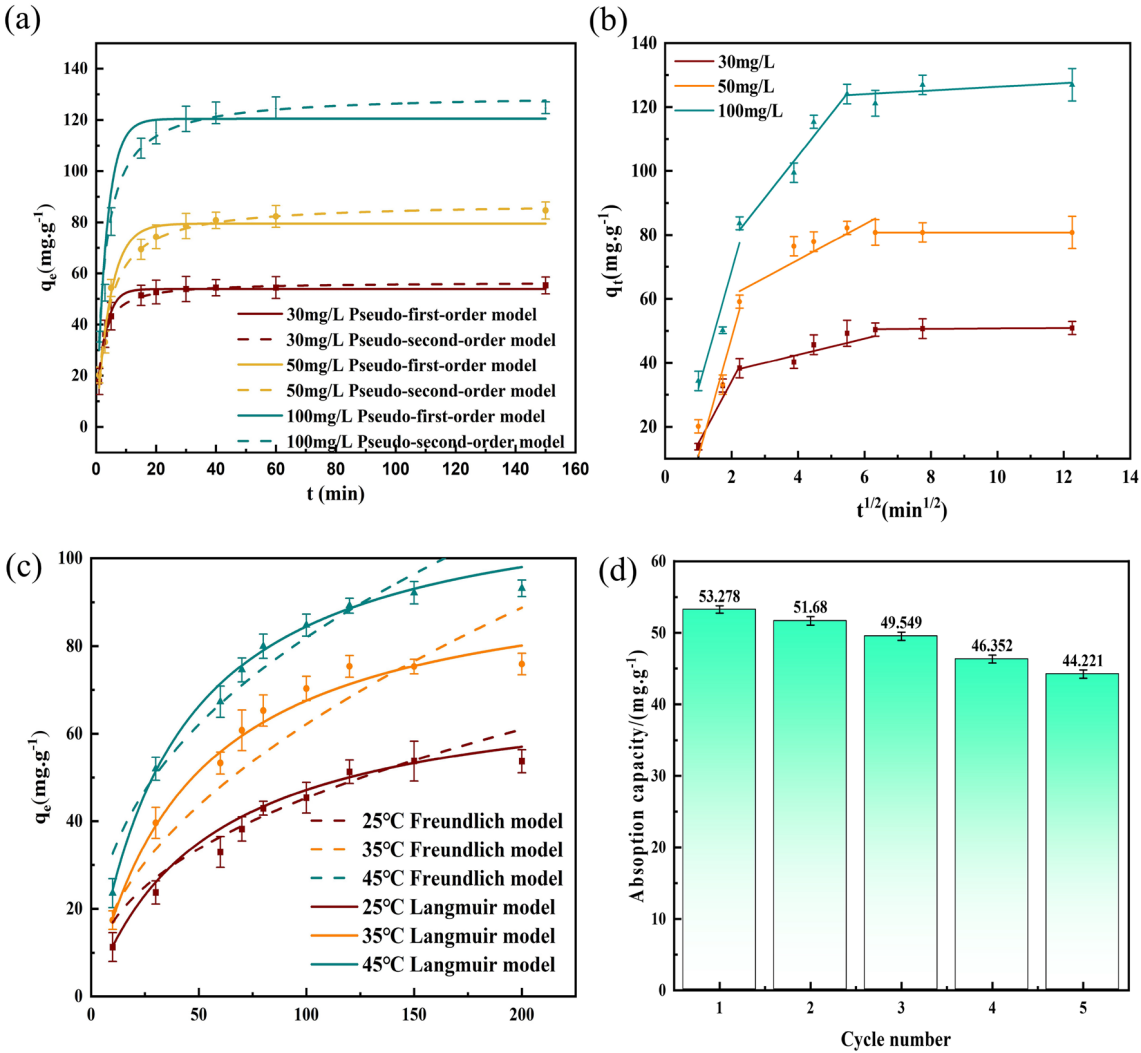
(**a**) Fit of the kinetic data with the pseudo-first-order model; (**b**) fit of the kinetic data with the pseudo-second-order model; (**c**) fit of kinetic data with the intraparticle diffusion model; (**d**) Isotherm model for MO adsorption by the Fe_3_O_4_@SiO_2_ nanomaterial;

The adsorption rate is a key parameter used to explore the performance of the adsorbent and elucidate the adsorption process, so the pseudo-first-order model, pseudo-second-order model and the intraparticle diffusion model were used to fit the experimental data and determine the process of MO adsorption by the Fe_3_O_4_@SiO_2_ nanomaterials; the fitted data are shown in Tables 4 and 5, and the fitted results are shown in Fig. 7a–c.

**Table 4:** Kinetic fitting parameters for adsorption of MO by the Fe_3_O_4_@SiO_2_ nanomaterial.

C_0_(mg/L)	Pseudo-first-order				Pseudo-second-order			
	K_1_ (1/min)	q_e.exp_ (mg/g)	R_1_^2^	ε%	K_2_ (g/mg min)	q_e.exp_ (mg/g)	R_2_^2^	ε%
30	0.359	53.845	0.985	2.732	0.010	56.559	0.989	1.223
50	0.214	79.515	0.971	6.356	0.003	87.669	0.986	2.378
100	0.288	120.433	0.976	7.298	0.003	130.018	0.995	0.523

**Table 5:** Intraparticle diffusion model for MO adsorption by the Fe_3_O_4_@SiO_2_ nanomaterial.

C_0_(mg/L)	Intraparticle diffusion model								
	K_d1_ (mg/g min^1/2^)	C (mg/g)	R^2^	K_d2_ (mg/g min^1/2^)	C(mg/g)	R^2^	K_d3_ (mg/g.min^1/2^)	C (mg/g)	R^2^
30	19.357	4.424	0.946	2.525	32.449	0.903	0.064	50.144	0.911
50	37.016	26.113	0.843	9.521	30.368	0.809	4.108	80.768	0.963
100	36.752	4.717	0.889	10.017	64.371	0.719	0.652	120.572	0.905

The pseudo-first-order equation is^33^:

5 $$ q_{t} = q_{e} (1 - e^{{k_{1} t}} ) $$

The pseudo-second-order equation is^34^:

6 $$ q_{t} = \frac{{q_{e}^{2} k_{2} t}}{{1 + k_{2} q_{e} t}} $$

where q_e_ and q_t_ (mg/g) are the adsorption capacities at adsorption equilibrium and at time t (min), respectively; k_1_ (1/min) and k_2_ (g/mg.min) are the adsorption rate constants for the pseudo-first-order model and pseudo-second-order model, respectively, and were calculated from the slope of the linear fit; finally, t is the reaction time (min).The kinetic model is chosen based on the deviation of q_e_ obtained from Eqs. 5 and 6 (calculated q_e_) and the q_e_ obtained from the experiment. Normalized standard deviation (ε%) is used to compare the applicability of either of these models. Commonly, the kinetic model which gives a smaller normalized standard deviation is used to describe the adsorption^35^ . The normalized standard deviation (ε %) is given by Eq. 7:

7 $$ \varepsilon \% = \left[ {\sqrt {\frac{{\Sigma [q_{t,\exp } - q_{t,cal} /q_{t,\exp } ]^{2} }}{n - 1}} } \right] \times 100\% $$

The intraparticle diffusion model was used to determine the rate-limiting step of the adsorption process. The expression for the intraparticle diffusion model is shown in Eq. 8^36^:

8 $$ q_{t} = K_{di} t^{1/2} + C $$

where k_di_ (mg.g^−1^·min^−1/2^) is the internal diffusion rate constant and C (mg g^−1^) is the thickness of the boundary layer.

Figure 7a,b and Table 4 show that for different MO concentrations, the correlation coefficient R^2^ of Pseudo-second-order model is higher and the range of relative deviation coefficients ε% is smaller for different concentrations of MO solutions, so the adsorption process of MO adsorption by Fe_3_O_4_@SiO_2_ nanomaterials is more consistent with pseudo-second-order model. To further confirm the kinetic behavior of anionic dye adsorption on Fe_3_O_4_@SiO_2_, an internal diffusion model was used to fit the adsorption data. From the fitted plots for the internal diffusion model in Fig. 7c, the MO adsorption process occurred in three stages for all three concentrations. The first stage involved diffusion of the MO molecules from the solution to the adsorbent surface, which corresponded to surface diffusion, and the adsorption rate was faster in this stage due to the high adsorption driving force. The second stage involved diffusion of the MO from the surface of the adsorbent to the inner surfaces of the mesopores, which corresponded to the internal diffusion process. The third stage involved slowing of the diffusion rate due to the low concentration of residual dye, and this constituted the adsorption equilibrium stage. Additionally, Table 5 shows that for the three different concentrations, K_d3_ < K_d2_ < K_d1_ and C ≠ 0, indicating that internal diffusion was the main, but not the only, rate-controlling step. The curve fits did not pass through the origin, indicating that diffusion from the boundary layer to the adsorbent surface cannot be neglected, so the adsorption of MO on the Fe_3_O_4_@SiO_2_ nanomaterial was determined by several rate-controlling steps^37^.

#### Adsorption isotherms

The Langmuir and Freundlich models were used to fit the adsorption data and analyze the adsorption process. The Langmuir model assumes single layer adsorption, in which the adsorbent molecules are in one-to-one correspondence with the adsorbent surface, and each occupied adsorption site cannot be used by another adsorbent molecule. Additionally, the odds that different sites on the adsorbent surface are used by the adsorbate are almost the same, and the Langmuir expression is shown below^16^ Eq. 9:

9 $$ q_{e} = \frac{{q_{m} K_{L} C_{e} }}{{1 + K_{L} C_{e} }} $$

where C_e_ (mg/L) is the concentration at adsorption equilibrium, q_e_ (mg/g) is the amount adsorbed when the adsorption process reaches equilibrium, K_L_(L/mg) is the Langmuir constant related to the adsorption binding energy, and q_m_ (mg/g) is the maximum amount of adsorbate on the adsorbent. K_L_ and q_m_ can be calculated by plotting against C_e_. There is also a parameter R_L_ in the Langmuir model, which can be used for determining the adsorption behavior and is defined by^38^ Eq. 10:

10 $$ R_{L} = \frac{1}{{1 + K_{L} C_{0} }} $$

where C_0_ is the actual concentration of the adsorbent solution. When 0 < R_L_ < 1, adsorption of the adsorbate occurs smoothly; when 1 < R_L_, the adsorption process is inhibited and when R_L_ = 0, adsorption does not occur.

The Freundlich model describes multilayer adsorption, the adsorption sites on the material surface are heterogeneous, and the expression is shown below^39^ Eq. 11:

11 $$ q_{e} = K_{F} C_{e}^{n} $$

where ${\text{K}}_{\text{F}}$ and n are the Freundlich constants, and the values are determined by fitting. When 2 < n < 10, adsorption occurs easily, and when n < 1, adsorption is difficult or some difficulties are encountered.

To choose between the Langmuir and Freundlich adsorption models, MO solutions with concentrations of 10, 30, 50, 70, 80, 90, 100,120 and 200 mg/L were added to 20 mg of the Fe_3_O_4_@SiO_2_ adsorbent at a constant temperature of 25 °C and stirred at 200 r/min and shaken. The absorbance was measured by sampling the supernatant after 30 min, and the isotherm fitting plots and data are shown in Fig. 7d and Table 6. In addition to the R^2^ table fitting accuracy, and were further evaluated by the root mean square error (RMSE), as shown in Eq. 12.

12 $$ RMSE = \sqrt {\frac{{\sum\limits_{i = 1}^{n} {\left( {X_{obs,i} - X_{{\text{model,i}}} } \right)^{2} } }}{n}} $$

**Table 6:** Adsorption isotherm parameters.

T(℃)	Langmuir				Freundlich			
	q_m_ (mg/g)	K_L_ (L/mg)	R^2^	RMSE	K_F_ (mg/g)/(mg/L)^n^	n	R^2^	RMSE
25℃	57.164	0.020	0.984	0.127	6.295	2.333	0.923	0.212
35℃	98.504	0.030	0.995	0.012	5.864	1.950	0.955	0.139
45 °C	116.624	0.053	0.996	0.005	12.938	2.495	0.949	0.177

As seen from Table 6**,** the Langmuir isothermal model provided better fits at all three temperatures, so the Langmuir isothermal adsorption model better described the adsorption of MO on the Fe_3_O_4_@SiO_2_ nanomaterial, and the process involved monolayer adsorption. The saturation adsorption amount increased from 57.164 to 116.624 mg/g as the temperature was increased from 25 to 45 °C, which also proved that adsorption of MO on the Fe_3_O_4_@SiO_2_ nanomaterial was an endothermic process. In addition, the Freundlich constants n > 1 and ${\text{R}}_{\text{L}}$=0.963 < 1 are provided in Table 6, which indicated that adsorption of MO was favorable.

#### Comparison of adsorption by different adsorbents

As shown in Table 7, the Fe_3_O_4_@SiO_2_ nanomaterial exhibited strong adsorption, and the magnetic Fe_3_O_4_ nanoparticles showed a nanosize effect and adsorbed MO via surface electrostatic attraction, and the adsorption capacity was enhanced after modification with the SiO_2_ coating. In addition, the adsorption capacity of Fe_3_O_4_@SiO_2_ was significantly higher than those of other adsorbents, indicating that Fe_3_O_4_@SiO_2_ has potential for use in treating organic dye wastewaters.

**Table 7:** Adsorption isotherm parameters.

^Adsorption^ materials	qmax (mg/g)	pH	^Contact time^ (min)	References
CuO NPs	121.95	6.5	540	^40^
PMOS	56.62	6.5	50	^41^
Fe2O3–BC	46.6	3	90	^42^
Carbon nanotubes	52.86	2	45	^34^
Fe3O4@SiO2	182.503	2	30	Present study

#### Adsorption thermodynamics

The adsorption thermodynamics indicate whether energy is released or absorbed during a reaction. To explore the mechanism for adsorption of MO on the Fe_3_O_4_@SiO_2_ nanomaterial, the adsorption data were analyzed thermodynamically at three different temperatures, 25 °C, 35 °C and 45 °C, to simulate the enthalpy and entropy changes. The thermodynamic parameters were the Gibbs free energy (△G^0^, KJ/mol), the enthalpy of absorption and (△H^0^, KJ/mol) and the entropy of absorption (△S^0^, J/(mol. K)). where since K_L_ has a magnitude in L/mg and the equilibrium constant Kc is dimensionless^43^, Eq. 15 was used for the transformation. and these are related as shown below.

13 $$ \Delta G^{0} = - RT\ln k_{c} $$

14 $$ \ln k_{c} = \Delta S^{0} /R - \Delta H^{0} /RT $$

15 $$ K_{C} = 10^{6} K_{L} $$

where R is the molar gas constant, 8.3145 J mol^−1^K^−1^; T is the absolute temperature; K_L_ is Langmuir’s constant and the equilibrium constant Kc is a dimensionless parameter; Qe is the equilibrium adsorption volume; and Ce is the equilibrium adsorption concentration.

The calculated thermodynamic parameters are shown in Table 8. When △H^0^ > 0, the reaction is endothermic; when △H^0^ < 0, the reaction is exothermic; △S^0^ > 0 indicates that the reaction increases the entropy and △S^0^ = 0 indicates that the reaction is at equilibrium. In addition, if △G^0^ < 0, the reaction proceeds spontaneously; if △G^0^ = 0, the reaction is at equilibrium and if △G^0^ > 0, the reaction cannot proceed spontaneously. From the data in the table, we can see that the positive value of △S^0^ indicates that the randomness increases at the solid–liquid interface during the adsorption of the three anionic dyes in aqueous solution onto the adsorbent,△G^0^ was negative and decreased with increasing temperature, which indicated that the adsorption process was spontaneous; also, △H^0^ was positive, which indicated the adsorption process was endothermic and higher temperatures favored adsorption; this was consistent with the results for isothermal adsorption line fitting.

**Table 8:** Thermodynamic parameters for MO adsorption by the Fe_3_O_4_@SiO_2_ nanomaterial.

T(K)	K_L_	Kc	△G^0^	△H^0^	△S^0^
	L/mg		(KJ/mol)	(KJ/mol)	(J/(mol K))
298	0.020	20,435.751	− 24.591	1.2417	4.2065
308	0.030	30,422.160	− 26.434		
318	0.053	53,475.070	− 28.784		

#### Reusability study

Regeneration experiments are important for probing reuse or recovery of an adsorbent. The experimental results shown in Fig. 7d indicated that after five desorption-adsorption cycles, the adsorption amount decreased from 53.278 to 44.221 mg/g, and the adsorption capacity was reduced, which may be attributed to incomplete desorption of the adsorbed MO from some of the adsorption sites. However, the amount adsorbed exceeded 80% of the initial amount adsorbed, so the Fe_3_O_4_@SiO_2_ nanomaterial was effectively recycled.

#### Adsorption of MO in real water samples

After treating as described in Sect. “Real Sample Preparation”, the results are shown in Table 9, from which it can be seen that the q_e_ of Fe_3_O_4_@SiO_2_ was 47.382 and 46.897 mg g^−1^ in the 100 mg/L MO solution configured for the water samples of FJ and KD, respectively, and the desorption rates were 78% and 80%, which were decreased, but the adsorption and desorption performances were still excellent, which proves that Fe3O4 can be used for the adsorption of the MO dye effluent from the real water samples.

**Table 9:** Adsorption of MO in different water samples on Fe_3_O_4_@SiO_2_ in real water samples.

Water samples	Added/(mg L^−1^)	Q/(mg g^−1^)	D (%)
Deionized water	100	53.882	83
FJ-SJ	100	47.382	78
KD-SJ	100	46.897	80

#### Exploration of the adsorption mechanism

**Figure 8:**
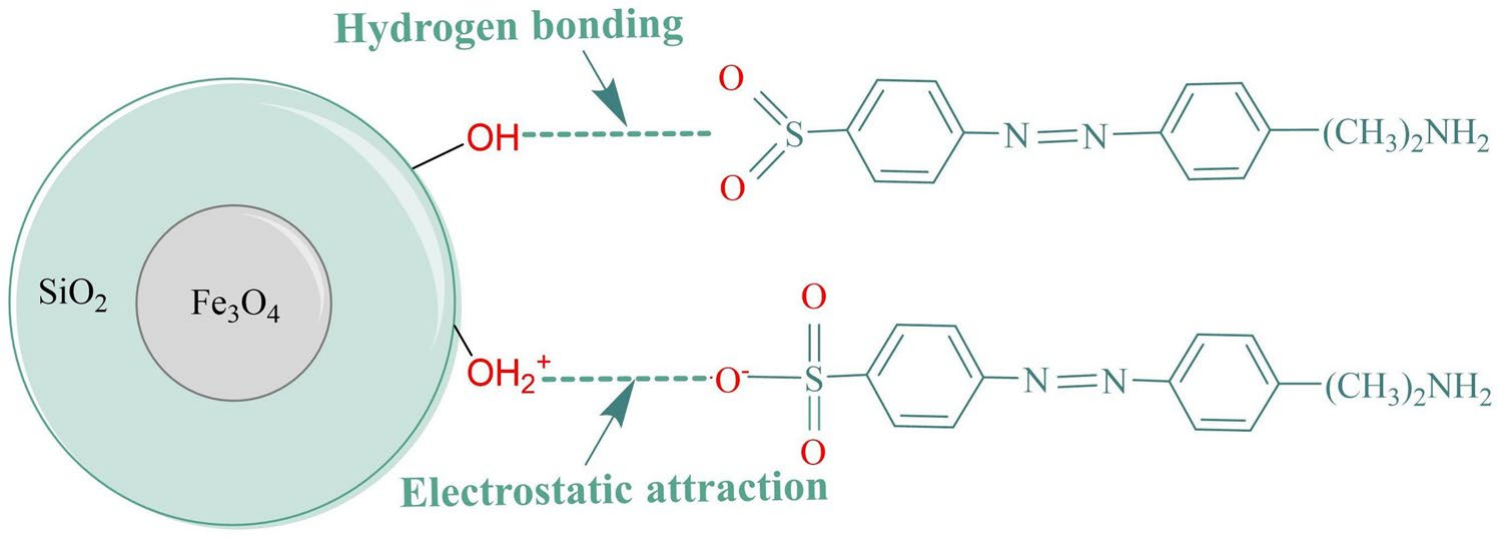
The adsorption mechanism of Fe_3_O_4_@SiO_2_ for MO.

According to the previous analysis, the increases in the average particle size and surface area of the Fe_3_O_4_@SiO_2_ nanomaterial formed more adsorption sites and increased the adsorption capacity. The physical properties of the Fe_3_O_4_@SiO_2_ indicated that the ferromagnetic nanoparticles on the surface of the material provided a good magnetic response, and this enabled fast and controlled separation of Fe_3_O_4_@SiO_2_ with an applied magnetic field to improve the adsorption efficiency. Second, the mesoporous structure of the Fe_3_O_4_@SiO_2_ nanomaterial had an important impact on adsorption. Fe_3_O_4_@SiO_2_ nanomaterials usually have highly ordered pore structures, and these pores provide larger surface areas and more adsorption sites, and the pore sizes can be tuned to achieve efficient adsorption of MO. For chemisorption of MO on Fe_3_O_4_@SiO_2_, the appearance of amorphous SiO_2_ in the XRD pattern and S, Na, and N in the EDS spectra and XPS data indicated successful adsorption of MO on the Fe_3_O_4_@SiO_2_. The FT-IR spectrum of Fe_3_O_4_@SiO_2_-MO showed a shift in the position of the –OH vibrational band at 3428 cm^−1^, which indicated hydrogen bonding and electrostatic interactions between the MO and Fe_3_O_4_@SiO_2_. Additionally, the C–O and H–O-C binding energies were shifted in the XPS spectra, indicating the involvement of –OH on the Fe_3_O_4_@SiO_2_ surface in the adsorption process. This was also confirmed by the pH experiments. Electrostatic interactions also played important roles in the adsorption of MO by Fe_3_O_4_@SiO_2_. The pH of the solution was altered to control the charge on the adsorbent surface, which affected the interactions between the adsorbent and the dye molecules, so hydrogen bonding and electrostatic interactions were involved in adsorption of MO by Fe_3_O_4_@SiO_2_, and the –OH groups played an important role in the adsorption process. The adsorption mechanism is shown in Fig. 8.

## Conclusions

In this thesis, Fe_3_O_4_@SiO_2_ core–shell nanomaterials with good adsorption capacities were obtained by using sol–gel chemistry to encapsulate the magnetic Fe_3_O_4_ with mesoporous SiO_2_, and the increased adsorption efficiency was attributed to the increased surface area of the Fe_3_O_4_@SiO_2_. The results showed that the highest MO adsorption capacity for Fe_3_O_4_@SiO_2_ was 182.503 mg/g, far more than that for unmodified Fe_3_O_4_, 15.5 mg/g. The adsorption data for the adsorbent were fitted with the pseudo-second-order kinetic model, and the intraparticle diffusion kinetics indicated that the adsorption process was determined by multiple rate-controlling steps. Thermodynamic studies showed that the adsorption of MO on Fe_3_O_4_@SiO_2_ was endothermic and spontaneous, and the MO interacted with the adsorbent via electrostatic interactions and hydrogen bonding. In addition, Fe_3_O_4_@SiO_2_ showed good regeneration ability, and the adsorption capacity reached 83% of the initial adsorption capacity after 5 reuse cycles. Fe_3_O_4_@SiO_2_ core–shell nanomaterials for treating real water samples still have superior unit adsorption and desorption rates .Therefore, the Fe_3_O_4_@SiO_2_ nanomaterial exhibited good adsorption performance and was safe, efficient and reusable, so it is an ideal adsorbent for treating MO dye wastewater.

### Supplementary Information

Supplementary Figures.

## Data Availability

Data will be made available on request. If anyone would like to receive data from this study, please contact Huanhuan Jin at 3,518,623,645@qq.com.
