# Supplementary material for: Magnetic Fe3O4@SiO2 study on adsorption of methyl orange on nanoparticles
Source: Sci Rep. 2024 Jan 12;14:1217. doi: 10.1038/s41598-023-50368-x (PMC10786890; doi:10.1038/s41598-023-50368-x)
Supplement: Supplementary file 1 — Supplementary Figures. [file 41598_2023_50368_MOESM1_ESM.docx]

**Supplementary materials**


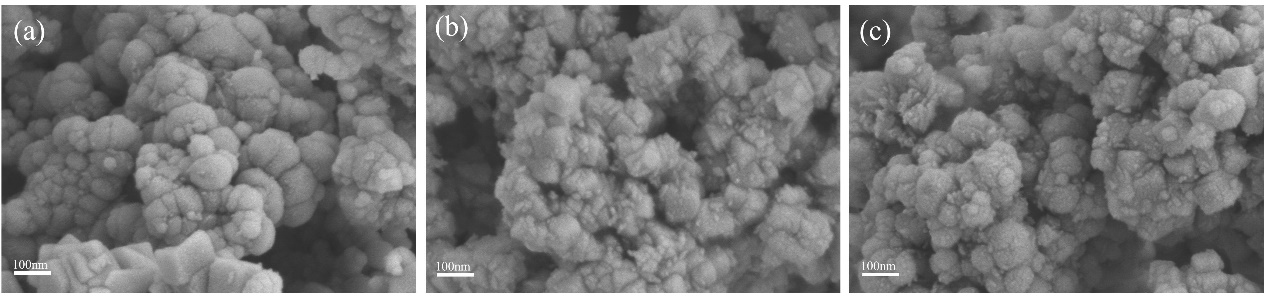


**Fig.S1** (a), (b), and (c) SEM images of Fe_3_O_4_, Fe_3_O_4_@SiO_2,_ and Fe_3_O_4_@SiO_2_-MO, respectively;


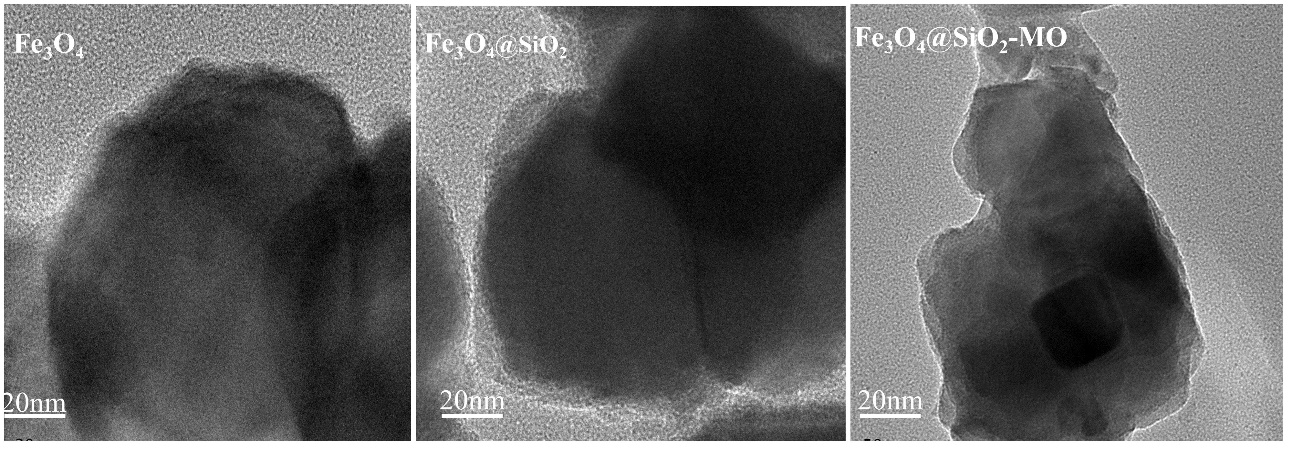


**Fig.S2** TEM images of Fe_3_O_4_, Fe_3_O_4_@SiO_2_ and Fe_3_O_4_@SiO_2_-MO.
